# Taxonomy and phylogeny of *Smaragdinisetamusae* sp. nov. and *Albifimbriaverrucaria* (Hypocreales, Stachybotryaceae) on *Musa* from Thailand

**DOI:** 10.3897/BDJ.10.e89360

**Published:** 2022-07-27

**Authors:** Binu C. Samarakoon, Dhanushka N. Wanasinghe, Jayarama Bhat, Putarak Chomnunti

**Affiliations:** 1 Center of Excellence in Fungal Research, Mae Fah Luang University, Chiang Rai, 57100, Thailand Center of Excellence in Fungal Research, Mae Fah Luang University Chiang Rai, 57100 Thailand; 2 School of Science, Mae Fah Luang University, Chiang Rai, 57100, Thailand School of Science, Mae Fah Luang University Chiang Rai, 57100 Thailand; 3 CIFOR-ICRAF China Program, World Agroforestry (ICRAF), Kunming 650201, China CIFOR-ICRAF China Program, World Agroforestry (ICRAF) Kunming 650201 China; 4 Centre for Mountain Futures (CMF), Kunming Institute of Botany, Chinese Academy of Sciences, Honghe 654400, Yunnan, China Centre for Mountain Futures (CMF), Kunming Institute of Botany, Chinese Academy of Sciences Honghe 654400, Yunnan China; 5 Department of Botany, Goa University, Goa, India Department of Botany, Goa University Goa India; 6 School of Science, Mae Fah Luang University, Chiang Rai 57100, Thailand School of Science, Mae Fah Luang University Chiang Rai 57100 Thailand

**Keywords:** one new species, banana, fungi, host, hyaline conidia, Musaceae, myrothecium-like, saprobes

## Abstract

**Background:**

*Smaragdinisetamusae* is introduced as a leaf-based novel saprobic species from *Musa*. Multi-gene phylogenetic analyses of internal transcribed spacer (ITS), RNA polymerase II second largest subunit (rpb2) and β-tubulin (tub2) data support the taxonomic placement of the new collection in *Smaragdiniseta* (Hypocreales, Stachybotryaceae). The novel species is characterised by cup-shaped sporodochia covered by numerous peripheral setae and simple hyaline, guttulate conidia produced by the ultimate branches (phialides) of conidiophores.

**New information:**

This is the first report of *Smaragdiniseta* from Thailand and on Musaceae. In addition, we report *Albifimbriaverrucaria* for the first time from Thailand, based on morpho-molecular evidence.

## Introduction

Crous et al. (2014) established Stachybotryaceae and Lombard et al. (2016) further revised the family, based on phenotypic characteristics and molecular analyses of LSU, ITS, rpb2, cmdA, tef1 and tub2 sequences. These arguments were accepted in the recent classifications of Sordariomycetes by Hyde et al. (2020) and Wijayawardene et al. (2020). The polyphyletic nature of *Myrothecium* (Chen et al. 2016) was further justified by Lombard et al. (2016) who established 13 new genera in Stachybotryaceae with myrothecium-like morphology viz. *Albifimbria*, *Capitofimbria*, *Dimorphiseta*, *Gregatothecium*, *Inaequalispora*, *Myxospora*, *Neomyrothecium*, *Paramyrothecium*, *Parvothecium*, *Smaragdiniseta*, *Striaticonidium*, *Tangerinosporium* and *Xenomyrothecium*. *Smaragdiniseta* was established to accommodate *S.bisetosa* (=*Myrotheciumbisetosum*), which is characterised by cup-shaped sporodochia with straight, hyaline, smooth-walled peripheral setae (Rao and De Hoog 1983, Lombard et al. 2016). The setae grow rapidly from sporodochia and are soon covered by a weft of emerald green, echinulate marginal hyphae (Rao and De Hoog 1983). The sexual morph of *Smaragdiniseta* is yet to be determined.

The taxonomic placement of *M.bisetosum* was doubted by Rao and De Hoog (1983) as it is morphologically resembling *Sarcopodium* by the formation of setae (Ehrenberg 1818). However, Rao and De Hoog (1983) distinguished *M.bisetosum* from *Sarcopodium* by the macroscopic colour of the conidiomata. They interpreted the new collection that was similar in morphology to *Sarcopodium* and *Myrothecium*. In the phylogenetic analyses of Lombard et al. (2016), *M.bisetosum* formed a monophyletic lineage sister to *Albifimbria* that was well separated from *Myrothecium*. *Smaragdiniseta* remains monospecific (Index Fungorum 2022, Cooper and Kirk 2022) and no additional taxonomic work has been conducted since Lombard et al. (2016).

In Lombard et al. (2016), the ex-neotype strain of *Myrotheciumverrucaria* formed another highly supported monophyletic clade in Stachybotryaceae. *Albifimbria* was introduced to classify this lineage and *M.verrucaria* was synonymised and typified under *Albifimbria*. In addition, *A.lateralis*, *A.terrestris* and *A.viridis* were introduced as novel taxa (Lombard et al. 2016). *Albifimbria* is characterised by the production of verrucose setae around the sporodochia (Lombard et al. 2016). In addition, some *Albifimbria* species bear funnel-shaped mucoid appendages in conidia (Lombard et al. 2016). Currently, four species of *Albifimbria* are listed in Index Fungorum (Cooper and Kirk 2022).

We are studying the saprobic fungi associated with *Musa* spp. from Thailand with the intention of providing a better understanding of their taxonomy, based on both morphology and phylogeny (Samarakoon et al. 2020a, Samarakoon et al. 2020b, Samarakoon et al. 2021a, Samarakoon et al. 2021b). This study is aimed at documenting two myrothecium-like taxa in Stachybotryaceae isolated from the dead leaves of *Musa*. Based on morphological illustrations, descriptions and phylogenetic analyses, we introduce one of our collections as *Smaragdinisetamusae* sp. nov. from Mae Sai, Chiang Rai, Thailand. This is the second species in *Smaragdiniseta* which further validates the taxonomic establishment of Lombard et al. (2016) and breaks the monotypic nature of the genus. In addition, we report *Albifimbriaverrucaria* on *Musa* sp. as a new country record to Thailand.

## Materials and methods

**Sample collection, morphological studies and isolation**

Dead leaves of *Musa* with characteristic sporodochia were collected from Thailand from January to October 2019. Specimens were transferred to the laboratory in small cardboard boxes. Fungi were observed using a Motic SMZ 168 series microscope (Motic Asia, Kowloon, Hong Kong). Conidiomata were mounted on glass slides in tap water and lactoglycerol for examination and photomicrography. The specimens were further observed using a Nikon ECLIPSE 80i compound microscope (Nikon Instruments Inc., Melville, NY, USA) and photographed using a Canon 550D digital camera (Canon Inc., Ota, Tokyo, Japan). Measurements were taken with the aid of Tarosoft (R) Image Frame Work programme. More than 10 measurements were made for the structures. The images were further arranged using Adobe Photoshop CS6 Extended version 10.0 software (Adobe Systems, USA).

Single spore isolations for the samples were conducted according to Senanayake et al. (2020). Germinated conidia were individually transferred to potato dextrose agar (PDA) plates and incubated at 25°C. Colony characters were examined after two weeks. Dried herbarium specimens were deposited in the Mae Fah Luang University Herbarium (Herb. MFLU), Chiang Rai, Thailand. Living cultures in PDA were deposited in the Culture Collection of Mae Fah Luang University (MFLUCC). Facesoffungi numbers (Jayasiri et al. 2015) and MycoBank numbers (http://www.MycoBank.org) were received for the isolates. The illustrations and descriptions were submitted to the GMS MICROFUNGI (gmsmicrofungi.org) database (Chaiwan et al. 2021). The finalised alignment and tree were submitted to Zenodo (https://zenodo.org/record/6867700#.Ytghz3ZBzIU).

**DNA extraction, PCR amplification and sequencing**

DNA was extracted from the mycelium of 14 days-old cultures. The mycelium was crushed using a plastic pestle and DNA was extracted using Biospin Fungus Genomic DNA Extraction Kit-BSC14S1 (BioFlux, P.R. China) following the manufacturer's guidelines. Three gene regions; viz. internal transcribed spacer (ITS), partial β-tubulin (tub2) and partial second largest subunit of the DNA-directed RNA polymerase II (rpb2), were amplified using ITS5/ITS4 (White et al. 1990), Bt2a and Bt2b (Glass and Donaldson 1995) and fRPB2-5f/fRPB2-7cR (Liu et al. 1999), respectively.

Polymerase chain reaction (PCR) was conducted using the following protocol. The total volume of the PCR reaction was 25 μl and comprised 12.5 μl of 2 × Power Taq PCR MasterMix (a premix and ready-to-use solution, including 0.1 Units/μl Taq DNA Polymerase, 500 μm dNTP Mixture each (dATP, dCTP, dGTP, dTTP), 20 mM Tris-HCL pH 8.3, 100 mM KCl, 3 mM MgCl_2_, stabiliser and enhancer), 1 μl of each primer (10 pM), 2 μl genomic DNA and 8.5 μl of deionised water. The total reaction comprised 35 cycles. The annealing temperatures were according to Lombard et al. (2016) and Samarakoon et al. (2021b). The amplified PCR fragments were sent to TsingKe Biological Technology (Beijing) Co., China for sequencing. DNA sequence data obtained were deposited in GenBank (https://www.ncbi.nlm.nih.gov/).

**Sequence alignment**

Newly-generated sequence data of different gene regions were subjected to BLAST searches using BLASTn in GenBank (https://blast.ncbi.nlm.nih.gov/Blast.cgi) to retrieve similar sequences. The results and initial morphology indicated that our strains belong to Stachybotryaceae (Hypocreales). The collection numbers for these similar sequences (Table 1) were then downloaded from GenBank, based on BLASTn results and Lombard et al. (2016). Single gene alignments were made by MAFFT v. 7.036 (http://mafft.cbrc.jp/alignment/server/large.html, Katoh et al. 2019) using the default settings and later refined as necessary using BioEdit v. 7.0.5.2 (Hall 1999).

**Phylogenetic analyses**

Maximum Likelihood (ML) trees were generated using the RAxML-HPC2 on XSEDE (8.2.8) (Stamatakis et al. 2008, Stamatakis 2014) in the CIPRES Science Gateway platform (Miller et al. 2010) with GTR+I+G model of evolution for single and multi-gene alignments. Bootstrap supports were gained by running 1000 pseudo-replicates. Maximum Likelihood (ML) bootstrap values equal to or greater than 60% are given above each node of the phylogenetic tree (Fig. 3).

A Bayesian analysis was conducted with MrBayes v. 3.1.2 (Huelsenbeck and Ronquist 2001) to evaluate posterior probabilities (Rannala and Yang 1996, Zhaxybayeva and Gogarten 2002) by Markov Chain Monte Carlo sampling (MCMC). Two parallel runs were conducted, using the default settings and with the adjustments as follows; four simultaneous Markov chains were run for 2,000,000 generations and trees were sampled every 100^th^ generation and 20000 trees were obtained. The first 4,000 trees of the burn-in phase were discarded. The remaining 16,000 trees were taken for calculating PP in the majority rule consensus tree. Branches with Bayesian posterior probabilities (BYPP) equal to or greater than 0.95 are written above each node of the multigene tree (Fig. 3). The trees were displayed with FigTree v.1.4.0 (Rambaut 2011) and re-arranged in Microsoft PowerPoint.

## Taxon treatments

### Smaragdiniseta musae

Samarakoon & Chomnunti sp. nov.

6562EC40-F30B-5243-97EB-CC8581761420

MB844095

https://www.facesoffungi.org/?s=FoF10846

#### Materials

- **Type status:** Holotype. **Occurrence:** occurrenceRemarks: Found on a dead leaf of *Musa* sp.; recordNumber: BNS264; recordedBy: Binu C. Samarakoon; disposition: Living cultures: MFLUCC 22-0015 and MFLUCC 22-0016; associatedSequences: GenBank MFLUCC 22-0015: ON563485 (ITS), ON586151 (rpb2), ON572191 (tub2); MFLUCC 22-0016:ON563486 (ITS), ON58615(rpb2), ON572192 (tub2); **Taxon:** scientificName: *Smaragdinisetamusae* Samarakoon & Chomnunti; kingdom: Fungi; phylum: Ascomycota; class: Sordariomycetes; order: Hypocreales; family: Stachybotryaceae; genus: Smaragdiniseta; specificEpithet: *musae*; taxonRank: species; scientificNameAuthorship: Samarakoon & Chomnunti; **Location:** continent: Asia; country: Thailand; stateProvince: Chiang Rai; locality: Mae Sai; **Identification:** identifiedBy: Binu C. Samarakoon; **Event:** year: 2019; month: June; day: 16; habitat: Terrestrial; **Record Level:** institutionID: MFLU; collectionID: MFLU 22-0047

#### Description

Saprobic on dead leaves of *Musa* sp. **Sexual morph**: Undetermined. **Asexual morph**: Sporodochia 0.2–0.35 mm diam., cup-shaped, scattered, solitary, circular with an entire margin, initially emerald green, later becoming black, composed of hyaline, erect conidiophores, surrounded by filamentous white marginal hyphae and setae at periphery. Fungal hyphae arranged in parallel like a palisade layer, compacted, verrucose, olivaceous-brown or hyaline, sometimes warticulate, septate, branched, irregularly thick-walled, with ultimate hyphal cell rounded at apex and flat at base; hyphal cells: 10–14 × 0.8–2.5 μm (x̄ = 13.3 × 1.6 μm, n = 30). Aggregated hyphae hyaline or emerald green when immature, tan brown at maturity. Setae fast growing from marginal hyphae, numerous, aggregating as a pale grey brush, elongated, straight or slightly curved, tapering towards apex, hyaline, septate, unbranched, apiculate at apex, sometimes caudate, truncate or rounded, swollen to globose to ovate or rounded at base, setae wall 0.4–1.5 μm (x̄ = 0.8 μm, n = 30) thick, often smooth, sometimes rough with hyaline acellular coatings, (60–)90–160(–250) × 0.8–2.8 μm (x̄ = 126.4 × 1.6 μm, n = 30); septa 4–8 μm apart. Marginal hyphae often coiling or growing around the setae or projecting out, forming a wefty cover around setae, 95–125 μm (x̄ = 106 μm, n = 30) wide. Conidiophores 4–14 × 1.3–2.5 μm (x̄ = 12.9 × 1.7 μm, n = 30), macronematous, hyaline, smooth, thin-walled, arising from sub hyaline or pale brown, with slightly thickened and swollen basal cells, often with a narrow truncate base, wider in the middle, tapering to rounded at apex, 3–4 × 5–6 μm (x̄ = 3.5 × 5.5 μm, n = 10), septate, rarely unbranched, mostly branched and with 2–3 conidiogenous sub-branches at each node. Conidiogenous cells phialidic, rough or thin-walled, rod-shaped, elongated or ovate, 4–9 × 1–3 μm (x̄ = 6.8 × 1.9 μm, n = 20), without a collarette, pinching off simple conidia at the apex of each phialide. Conidia simple, hyaline, smooth, thin-walled, elliptic or slightly ovate, rounded at one end and acute at other end, with two distinct guttules at vertical ends, sometimes with 3–5 guttules, 6–10 × 2–3.5 μm (x̄ = 8.3 × 2.7 μm, n = 10).

**Culture characteristics.** Conidia germinating on PDA after 48 hours, germ tubes being produced from the acute end. Colonies growing on PDA reaching 20 mm diam. after 2 weeks in light conditions at 25°C, mycelium mostly immersed, not slimy, cottony, pinkish-white, dense in the middle and comparatively sparse at the periphery. Radially and unevenly striated, colonies have a slightly wrinkled appearance from the top. The formation of sporodochia was not observed in mature colonies.

#### Etymology

The species epithet reflects the host genus, *Musa*.

#### Notes

**Figure 1.**
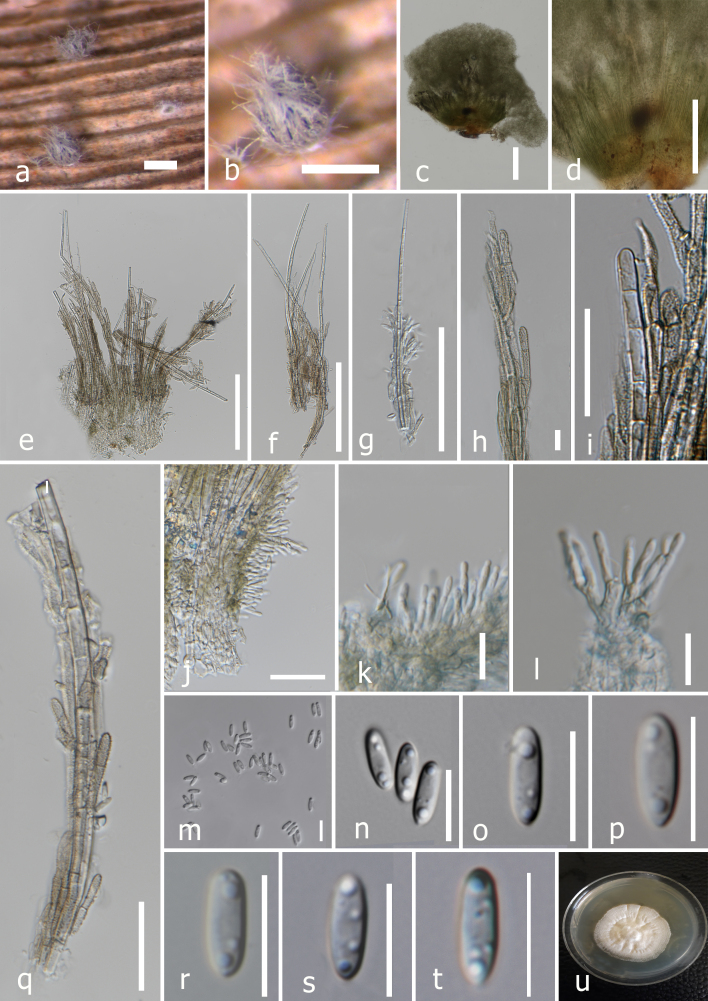
*Smaragdinisetamusae* (MFLU 22-0047, holotype) **a, b** sporodochia on the host; **c, d** cupulate sporodochia; **e-i, q** setae and marginal hyphae; **j-l** attachments of conidiophores and phialides; **m-t** conidia; **u** colony on PDA after 8 weeks. Scale bars: 400 μm (**a, b**), 100 μm (**c, d, i, q**), 50 μm (**e-h**), 20 μm (**j**), 10 μm (**k-t**).

Based on BLASTn search results of ITS, tub2 and rpb2 sequence data, *Smaragdinisetamusae* (Fig. 1) showed a high percentage identity (ITS = 97.23%, tub2 = 89.16% and rpb2 = 91.10%) without gaps to *S.bisetosa* (CBS 459.82). In the multigene phylogeny, *S.musae* clustered with *S.bisetosa* in having strong statistical support (100% ML, 1.00 BYPP) (Fig. 3). The base pair comparison of ITS, tub2 and rpb2 of our new taxon revealed 3.14% (17/540), 12.36% (35/283) and 9.34% (63/674) nucleotide differences with *S.bisetosa*. Besides, *S.musae* differs from *S.bisetosa* by the conidial morphology. The conidia of *S.musae* have two distinct guttules at the vertical poles. In addition, some conidia bear minute guttules at the centre. However, the taxonomic illustration and description of *S.bisetosa* did not indicate the guttule formation in the conidia (Rao and De Hoog 1983). Moreover, the conidia of *S.bisetosa* are obclavate, narrowly ellipsoidal or rod-shaped, whereas our new taxon has elliptic or slightly ovate-shaped conidia with a rounded top and an acute base. Both ends of the conidia of *S.bisetosa* are rounded or sometimes are found with a truncate base (Rao and De Hoog 1983). We have not observed a truncate base in the conidia of *S.musae*. Furthermore, the marginal hyphae of *S.musae* often coil or grow around the setae, but have never overgrown. The marginal hyphae always reach around 95–125 μm of the setae and terminate at a point. According to the description of Rao and De Hoog (1983), in *S.bisetosa*, the marginal hyphae always covered the entire setae. Our new collection is similar in morphology to the other genera in Stachybotryaceae in having conidiophores where the ultimate branches become phialides (Lombard et al. 2016). This feature phenotypically justifies the placement of our new collection in Stachybotryaceae. Based on distinct morphological characteristics and strong statistical support from our molecular phylogeny, *Smaragdinisetamusae* is, therefore, herein introduced as a new species on *Musa* sp. from Chiang Rai Province, Thailand. This is the first report of *Smaragdiniseta* on Musaceae from Southeast Asia. In addition, *S.musae* is the second taxon that is being described in this genus.

### Albifimbria verrucaria

(Alb. & Schwein.) L. Lombard & Crous

36DD5C21-38E3-578C-8CE1-7DBBE5E32C09

https://doi.org/10.3767/003158516X691582

MB815927

https://www.facesoffungi.org/?s=FoF04192

#### Materials

- **Type status:** Other material. **Occurrence:** occurrenceRemarks: Found on a dead leaf of *Musa* sp.; recordNumber: BNS292; recordedBy: Binu C. Samarakoon; disposition: Living culture: MFLUCC 22-0017; associatedSequences: GenBank: MFLUCC 22-0017: ON563487(ITS), ON586153(tub2); **Taxon:** scientificName: *Albifimbriaverrucaria* (Alb. & Schwein.) L. Lombard & Crous; kingdom: Fungi; phylum: Ascomycota; class: Sordariomycetes; order: Hypocreales; family: Stachybotryaceae; genus: Albifimbria; specificEpithet: *verrucaria*; taxonRank: species; scientificNameAuthorship: (Alb. & Schwein.) L. Lombard & Crous; **Location:** continent: Asia; country: Thailand; stateProvince: Chiang Rai; municipality: Mae Sai; **Identification:** identifiedBy: Binu C. Samarakoon; **Event:** year: 2019; month: June; day: 25; habitat: Terrestrial; **Record Level:** institutionID: MFLU; collectionID: MFLU 22-0048

#### Description

Saprobic on dead leaves of *Musa* sp. **Sexual morph**: Undetermined. **Asexual morph**: Sporodochia cupulate or discoid, scattered or gregarious, having irregular or rounded outline composed of white marginal hypha, with conidial mass flattened or convex, pale olivaceous-green at an immature stage, black and shiny at maturity, 10–18 × 0.8–3 μm (x̄ = 12.3 × 2.4 μm, n = 20). Stroma rarely well-developed, usually with a thin layer of isodiametric or elongated hyaline cells 15–25 (x̄ = 16.8 μm, n = 10). Setae: not observed. Marginal hyphae hyaline, usually verrucose, septate, curling and coiling, some branched, rounded or blunt at apex, 1.5–4 (x̄ = 3.3, n = 20) in diam. Conidiophores arising from a thin stromatic layer, hyaline, smooth, 30–48 × 1–2 μm (x̄ = 42.2 × 1.7 μm, n = 30) septate, branching repeatedly, forming 2–4 branches at each level, with ultimate branches becoming phialides, which give rise to numerous conidia, conidiophores sometimes also arising from the hyphae. Phialides hyaline, rough-walled 30–48 × 1–2 μm (x̄ = 42.2 × 1.7 μm, n = 30), 3–7 in a whorl, closely packed in a dense parallel layer, cylindrical, hyaline, collate at the base, rounded or acute at apex, sometimes slightly tapering towards apex, 8–16 × 1–3.5 μm (x̄ = 12.2×1.8 μm, n = 30). Conidia broadly fusiform, always pointed at one end, mostly truncate or rounded at the other end, hyaline, sometimes sub hyaline, smooth, 5–9.5 × 2–3.5 μm (x̄ = 7.6 × 3.0 μm, n = 30).

**Culture characteristics.** Conidia germinated on PDA after 12 hours. Colonies growing on PDA reaching 40 mm diam. after 2 weeks in the light conditions at 25°C, mycelium is mostly immersed, not slimy, cottony, white, dense in the middle and comparatively sparse at the periphery, fast-growing. Sporodochia formed after 12 days at the centre as a black uneven ring.

#### Notes

**Figure 2.**
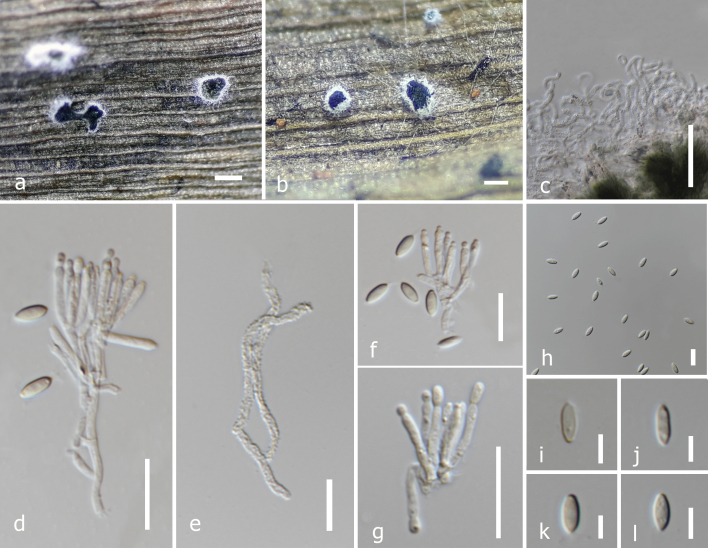
*Albifimbriaverrucaria* (MFLU 22-0048) **a, b** sporodochia on the host; **c, e** marginal hyphae; **d, f, g** conidiophores and phialides; **h-l** conidia. Scale bars: 400 μm (**a, b**), 100 μm (**i-j**), 50 μm (**c**), 10 μm (**d-h**), 5 μm (**i-l**).

**Figure 3.**
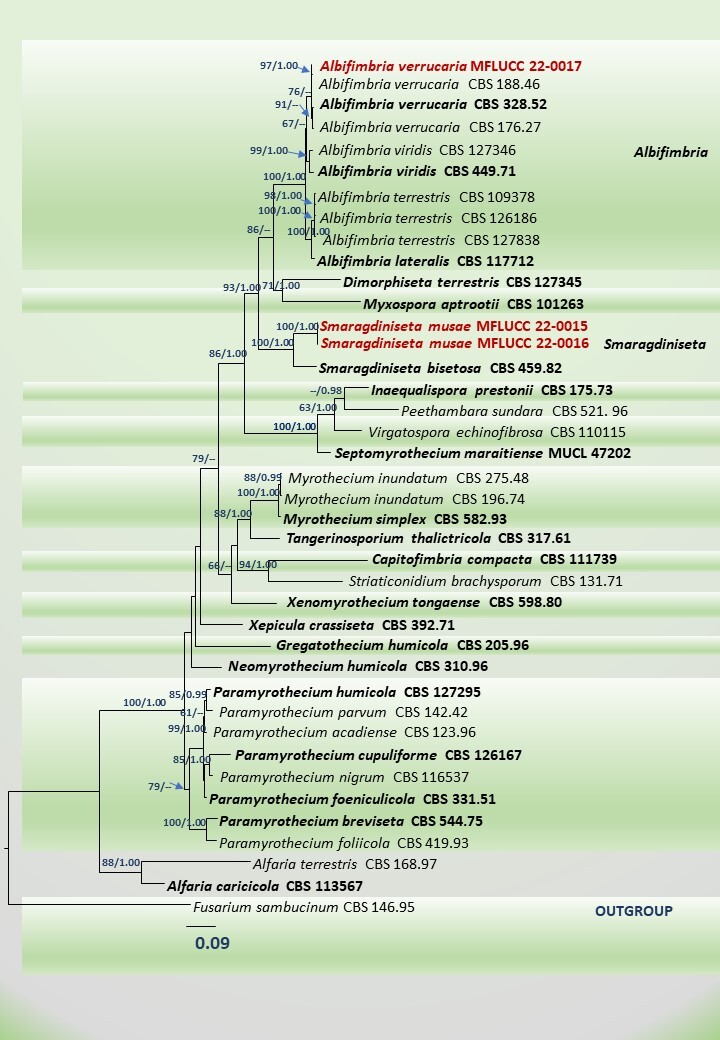
Maximum Likelihood tree revealed by RAxML analyses of ITS, rpb2 and tub2 sequence data of selected genera of Stachybotryaceae, showing the phylogenetic position of *Albifimbriaverrucaria* (MFLUCC 22-0017) and *Smaragdinisetamusae* (MFLUCC 22-0015, MFLUCC 22-0016). ML bootstrap supports (≥ 60%) and Bayesian posterior probabilities (≥ 0.95 BYPP) are given above the nodes, respectively. The tree is rooted with *Fusariumsambucinum* (CBS146.95) (Nectriaceae). Strains generated in this study are indicated in bold red. Ex-type strains are indicated in bold black. The scale bar represents the expected number of nucleotide substitutions per site.

Based on BLASTn search results of ITS, tub2 and rpb2 sequence data, our stain (MFLUCC 22-0017) showed a high similarity (ITS = 100% tub2 = 100% and rpb2 = 99%), excluding gaps to *Albifimbriaverrucaria* (CBS 188.46). In the multigene phylogeny, MFLUCC 22-0017 grouped with *A.verrucaria* strains with strong statistical supports (97% ML, 1.00 BYPP) (Fig. 3). Morphologically, our collection (Fig. 2) is similar to the descriptions of Tulloch (1972) and Lombard et al. (2016). *Albifimbriaverrucaria* has previously been reported from *Musa* sp. as a saprobe in Venezuela (Dennis 1970). This is the first report of *Albifimbria* from Thailand. MFLUCC 22-0017 is the first saprobic *A.verrucaria* strain found in Thailand. In addition, this is the second report of *Albifimbria* on Musaceae.

## Analysis

### Phylogenetic analyses

The combined ITS (1–599), rpb2 (604–1281) and tub2 (1286–1596) gene alignment was composed of 40 sequences that represented some of the selected taxa in Stachybotryaceae. The best scoring RAxML tree is presented (Fig. 3) with a final ML optimisation likelihood value of -13217.698. The matrix had 714 distinct alignment patterns with 15.47% of undetermined characters or gaps. Estimated base frequencies were: A = 0.239962, C = 0.278798, G = 0.255871, T = 0.225369; substitution rates AC = 1.609807, AG = 6.303653, AT = 1.525739, CG = 1.563045, CT = 10.838331, GT = 1.0; proportion of invariable sites I = 0.479596; gamma distribution shape parameter α = 0.654983. All trees (ML and BI) resulting from the multi-gene alignment were equal in topology, without notable differences from Lombard et al. (2016). *Smaragdinisetamusae* (MFLUCC 22-0015, MFLUCC 22-0016) formed an independent lineage sister to *S.bisetosa* (CBS 459.82) (ML = 100%, BYPP = 1.00) with strong statistical support. In addition, MFLUCC 22-0017 grouped with *Albifimbriaverrucaria* (CBS 188.46) (ML = 97%, BYPP = 1.00), respectively. RAxML trees generated from single gene allignments of ITS, rpb2 and tub2 sequences were attached as supplementray data (Suppl. material 1).

## Discussion

*Smaragdiniseta* has been previously documented as a saprobe only from terrestrial habitats (Rao and De Hoog 1983). There were no reports on pathogenic and endophytic lifestyles or sexual morphs that represent the genus. *Smaragdiniseta* was only discovered in India, while no other reports have been published on the occurrence worldwide. *Albifimbria* was subsequently discovered to show all three nutritional modes viz. as endophytes (Li et al. 2020, Wei et al. 2022), as pathogens (Gilardi et al. 2020, Herman et al. 2020, Rehman et al. 2021, Sharma et al. 2021) and as saprobes (Tulloch 1972). However, *Albifimbria* has mostly been discovered from plant hosts in terrestrial habitats (Tulloch 1972). In addition, the genus has also been reported in human blood (Masetti et al. 2020) and soil (Gurung et al. 2019, Murgia et al. 2019). Several genera of Stachybotryaceae, such as *Albifimbria*, *Memnoniella*, *Myrothecium* and *Stachybotrys*, are capable of producing bioactive compounds (Pervez et al. 2015, Wang et al. 2015, Li et al. 2020, Sun et al. 2020).

*Albifimbriaverrucaria* has been reported as a plant pathogen that causes stem necrosis and leaf spots on various crops, such as *Glycinelatifolia* (Herman et al. 2020), leafy vegetables (Matić et al. 2019, Rehman et al. 2021), ornamental crops (Matić et al. 2019) and tomato (Gilardi et al. 2020). In addition, *A.verrucaria* was also reported as an antagonistic agent on grapevine pathogens (Li et al. 2020). Additionally, *A.verrucaria* has been applied as a bio-pesticide to many weeds and nematodes (Assaf 2020). *Albifimbriaverrucaria* can produce many lytic enzymes (viz. lipase, protease and kinase) which can degrade the cuticles of insects and, thus, can be used as an insecticide (Assaf 2020, Weaver et al. 2021). Chemical screening of *Smaragdiniseta* isolates has not been conducted so far and still, the profiles remain undiscovered. Hence, apart from the taxonomic treatments, the chemical profiles of these genera also can be investigated as they are excellent sources of secondary metabolites. In addition, *A.verrucaria* was reported as a human pathogen causing keratomycosis (Moreno-Flores et al. 2020, Liu et al. 2021). Hence, there are opportunities for taxonomists to conduct sampling, isolation and identification of these hidden taxa from various hosts and provide baseline data for future research work.

## Supplementary Material

XML Treatment for Smaragdiniseta musae

XML Treatment for Albifimbria verrucaria

6EAC8D38-1116-5605-81CE-BB4CC66CB22A 10.3897/BDJ.10.e89360.suppl1 Supplementary material 1 Maximum Likelihood trees Data type Phylogenetic Brief description Maximum Likelihood trees revealed by RAxML analyses ITS, btub and rpb2 single gene regions File: oo_718103.docx https://binary.pensoft.net/file/718103 Binu C. Samarakoon

## Figures and Tables

**Table 1.** Names, culture collection numbers and their respective GenBank accession numbers of the Stachybotryaceae taxa that have been subjected to phylogenetic analyses. Type strains are superscripted with T and new collections are indicated in bold black.

**Species**	**Strain**	**GenBank Accession Numbers**		
		**ITS**	**rpb2**	**tub2**
*Albifimbrialateralis*	CBS 117712^T^	KU845881	KU845919	KU845957
*A.terrestris*	CBS 109378	KU845882	KU845920	KU845958
*A.terrestris*	CBS 126186	KU845883	KU845921	KU845959
*A.terrestris*	CBS 127838	KU845884	KU845922	KU845960
*A.verrucaria*	CPC 30056	KU845885	KU845923	KU845961
*A.verrucaria*	CBS 328.52^T^	KU845886	KU845924	KU845962
*A.verrucaria*	CBS 188.46	KU845888	KU845926	KU845964
***A.verrucaria***	**MFLUCC 22-0017**	**ON563487**	NA	**ON586153**
*A.viridis*	CBS 449.71^T^	KU845898	KU845936	KU845974
*A.viridis*	CBS 127346	KU845899	KU845937	KU845975
*Alfariacaricicola*	CBS 113567^T^	KU845983	KU846001	KU846014
*Alf.terrestris*	CBS 168.97	KU845987	KU846005	KU846018
*Capitofimbriacompacta*	CBS 111739^T^	KU846287	KU846349	KU846404
*Dimorphisetaterrestris*	CBS 127345^T^	KU846314	KU846375	KU846431
*Fusariumsambucinum*	CBS 146.95	KM231813	KM232381	KM232078
*Gregatotheciumhumicola*	CBS 205.96^T^	KU846315	KU846376	KU846432
*Inaequalisporaprestonii*	CBS 175.73^T^	KU846316	KU846377	KU846433
*Myrotheciuminundatum*	CBS 196.74^T^	KU846451	NA	KU846532
*M.simplex*	CBS 582.93^T^	KU846456	NA	KU846537
*Myxosporaaptrootii*	CBS 101263^T^	KU846458	KU846496	KU846539
*Neomyrotheciumhumicola*	CBS 310.96^T^	KU846467	KU846505	NA
*Paramyrotheciumacadiense*	CBS 123.96	KU846288	KU846350	KU846405
*Pa.breviseta*	CBS 544.75^T^	KU846289	KU846351	KU846406
*Pa.cupuliforme*	CBS 126167^T^	KU846290	KU846352	KU846407
*Pa.foeniculicola*	CBS 331.51^T^	KU846292	KU846354	KU846409
*Pa.foliicola*	CBS 419.93	KU846293	KU846355	KU846410
*Pa.humicola*	CBS 127295^T^	KU846295	KU846356	KU846412
*Pa.nigrum*	CBS 116537	KU846296	KU846357	KU846413
*Pa.parvum*	CBS 142.42	KU846297	KU846358	KU846414
*Peethambarasundara*	CBS 521.96	KU846470	KU846508	KU846550
*Septomyrotheciummaraitiense*	MUCL 47202^T^	NA	KU846510	NA
*Smaragdinisetabisetosa*	CBS 459.82^T^	KU847229	KU847281	KU847319
***S.musae***	**MFLUCC 22-0015^T^**	**ON563485**	**ON586151**	**ON572191**
***S.musae***	**MFLUCC 22-0016**	**ON563486**	**ON586152**	**ON572192**
*Striaticonidiumbrachysporum*	CBS 131.71	KU847230	KU847282	KU847320
*Tangerinosporiumthalitricola*	CBS 317.61^T^	KU847243	NA	KU847333
*Virgatosporaechinofibrosa*	CBS 110115	KU847244	KU847293	KU847334
*Xenomyrotheciumtongaense*	CBS 598.80^T^	KU847246	KU847295	KU847336
*Xepiculacrassiseta*	CBS 392.71^T^	KU847247	KU847296	KU847337

Abbreviations of culture collections; **CBS**: CentraalbureauvoorSchimmelcultures, Utrecht, The Netherlands, **CPC**: Working collection of Pedro Crous housed at CBS, **MFLUCC**: Mae Fah Luang University Culture Collection, Chiang Rai, Thailand, **MUCL**: Agriculture and Agri-Food Canada, Ottawa, Ontario, Canada, NA: Sequence data are not available in GenBank.
