# Supplementary material for: Taxonomy and phylogeny of Smaragdinisetamusae sp. nov. and Albifimbriaverrucaria (Hypocreales, Stachybotryaceae) on Musa from Thailand
Source: Biodivers Data J. 2022 Jul 27;10:e89360. doi: 10.3897/BDJ.10.e89360 (PMC9848465; doi:10.3897/BDJ.10.e89360)
Supplement: Supplementary material 1 — Maximum Likelihood trees [file bdj-10-e89360-s001.docx]

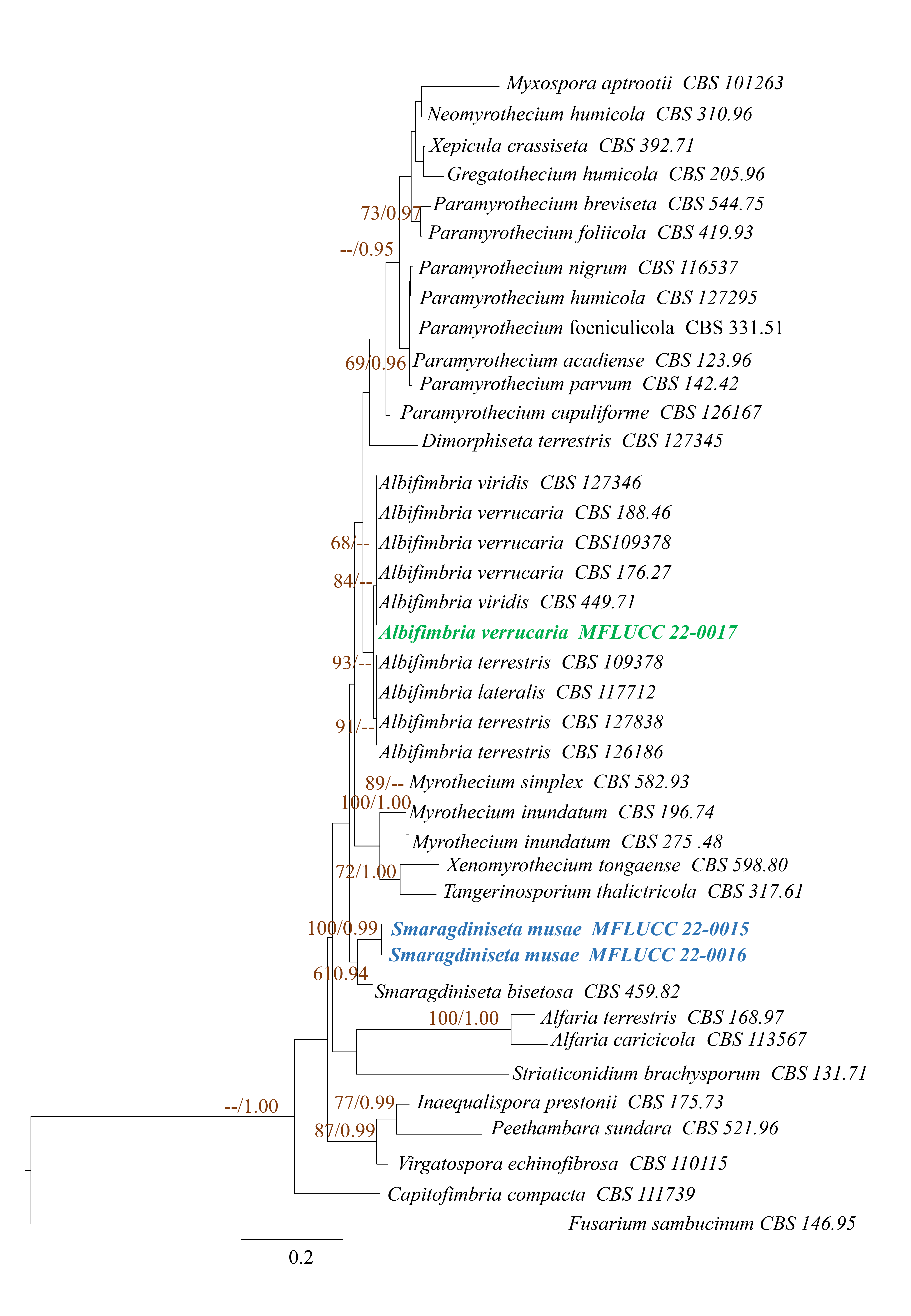


**Figure 1.** Maximum likelihood tree revealed by RAxML analyses of ITS sequence data of selected genera of Stachybotryaceae, showing the phylogenetic position of *Albifimbria verrucaria* (MFLUCC 22-0017) and *Smaragdiniseta musae* (MFLUCC 22-0015, MFLUCC 22-0016). ML bootstrap supports (≥60%) and Bayesian posterior probabilities (≥0.95 BYPP) are given above the nodes, respectively. The tree is rooted with *Fusarium sambucinum* (CBS146.95) (Nectriaceae). The scale bar represents the expected number of nucleotide substitutions per site.


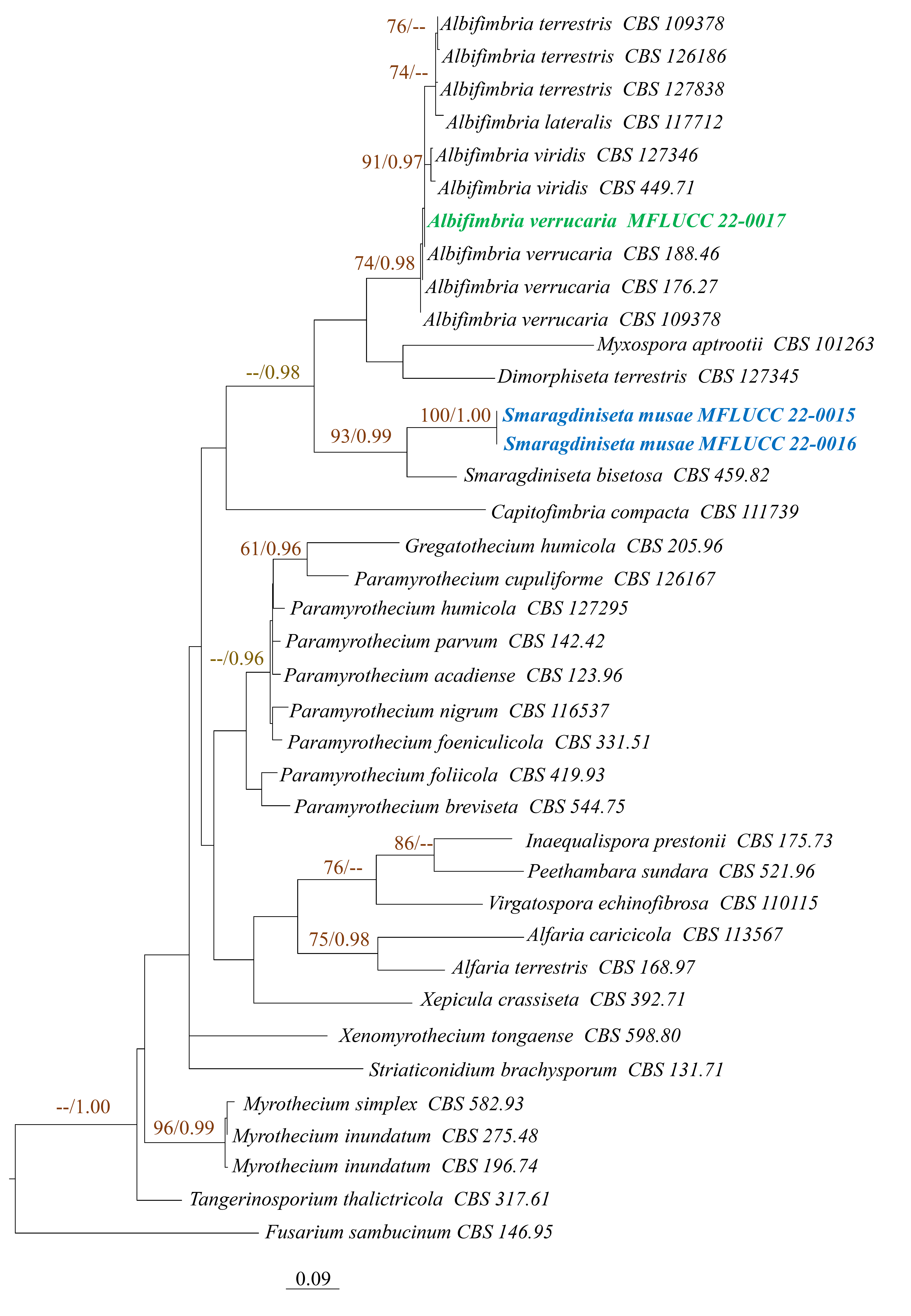


**Figure 2.** Maximum likelihood tree revealed by RAxML analyses of tub2 sequence data of selected genera of Stachybotryaceae, showing the phylogenetic position of *Albifimbria verrucaria* (MFLUCC 22-0017) and *Smaragdiniseta musae* (MFLUCC 22-0015, MFLUCC 22-0016). ML bootstrap supports (≥60%) and Bayesian posterior probabilities (≥0.95 BYPP) are given above the nodes, respectively. The tree is rooted with *Fusarium sambucinum* (CBS146.95) (Nectriaceae). The scale bar represents the expected number of nucleotide substitutions per site.


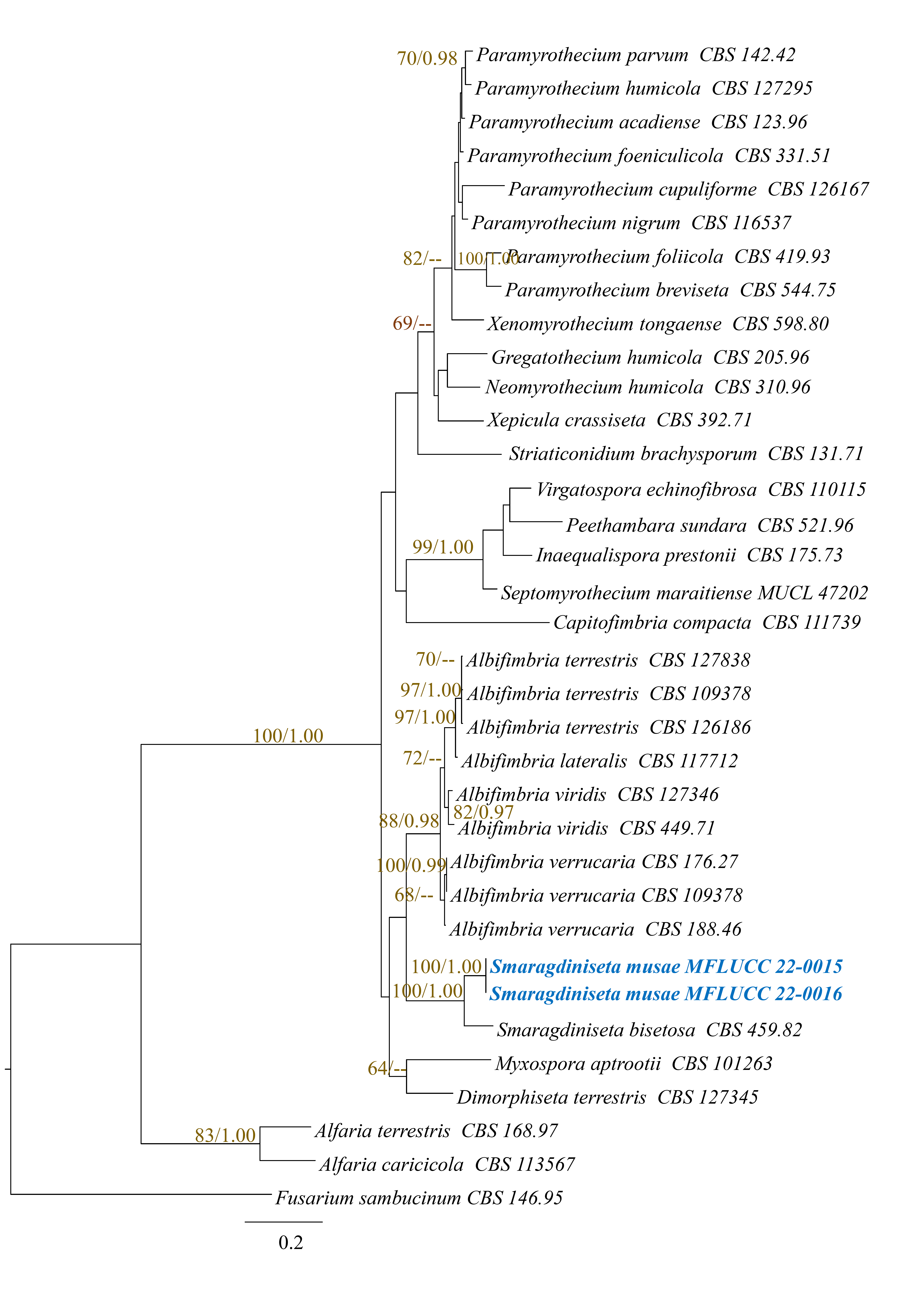


**Figure 3.** Maximum likelihood tree revealed by RAxML analyses of rpb2 sequence data of selected genera of Stachybotryaceae, showing the phylogenetic position of *Albifimbria verrucaria* (MFLUCC 22-0017) and *Smaragdiniseta musae* (MFLUCC 22-0015, MFLUCC 22-0016). ML bootstrap supports (≥60%) and Bayesian posterior probabilities (≥0.95 BYPP) are given above the nodes, respectively. The tree is rooted with *Fusarium sambucinum* (CBS146.95) (Nectriaceae). The scale bar represents the expected number of nucleotide substitutions per site.
